# Glioblastoma stem cell long non-coding RNAs: therapeutic perspectives and opportunities

**DOI:** 10.3389/fgene.2024.1416772

**Published:** 2024-07-02

**Authors:** Rasmani Hazra, Rinku Debnath, Arati Tuppad

**Affiliations:** ^1^ University of New Haven, Biology and Environmental Science Department, West Haven, CT, United States; ^2^ Department of Biotechnology, Indian Institute of Technology Madras, Chennai, India

**Keywords:** glioblastoma, cancer stem cells, glioblastoma stem cells, long non-coding RNAs, lncRNAs, therapeutic targets, 3D *in vitro* model

## Abstract

Glioblastoma poses a formidable challenge among primary brain tumors: its tumorigenic stem cells, capable of self-renewal, proliferation, and differentiation, contribute substantially to tumor initiation and therapy resistance. These glioblastoma stem cells (GSCs), resembling conventional stem and progenitor cells, adopt pathways critical for tissue development and repair, promoting uninterrupted tumor expansion. Long non-coding RNAs (lncRNAs), a substantial component of the human transcriptome, have garnered considerable interest for their pivotal roles in normal physiological processes and cancer pathogenesis. They display cell- or tissue-specific expression patterns, and extensive investigations have highlighted their impact on regulating GSC properties and cellular differentiation, thus offering promising avenues for therapeutic interventions. Consequently, lncRNAs, with their ability to exert regulatory control over tumor initiation and progression, have emerged as promising targets for innovative glioblastoma therapies. This review explores notable examples of GSC-associated lncRNAs and elucidates their functional roles in driving glioblastoma progression. Additionally, we delved deeper into utilizing a 3D *in vitro* model for investigating GSC biology and elucidated four primary methodologies for targeting lncRNAs as potential therapeutics in managing glioblastoma.

## 1 Glioblastoma and glioblastoma stem cells

Glioblastoma, classified as a grade IV glioma by the World Health Organization, is the epitome of complexity, lethality, and therapeutic resistance among brain tumors (Louis et al., 2007). Constituting 30% of all primary brain tumors and accounting for 80% of malignant cases, glioblastoma is the leading factor contributing to mortality among patients afflicted with primary brain tumors (Ostrom et al., 2023). The current standard of care for glioblastoma is multimodal, starting with maximal safe surgical resection, followed by postoperative radiation therapy and concurrent and adjuvant temozolomide (TMZ) administration (Khosla, 2016). Despite this comprehensive treatment approach, the median survival is a mere 14.6 months, with a 2-year overall survival rate of only 27%, and fewer than 5% of patients surviving beyond 5 years (Stupp et al., 2005; McNamara et al., 2014; Ostrom et al., 2023). The dismal prognosis for glioblastoma patients is primarily due to extensive intra-tumoral cellular heterogeneity, the induction of angiogenesis, uncontrolled cellular proliferation, and the diffuse infiltration of glioblastoma cells into the surrounding brain tissue, all of which contribute to a high recurrence rate (Aparicio-Blanco et al., 2020; Prager et al., 2020; Alves et al., 2021). Many of these characteristics can be attributed to glioblastoma stem cells (GSCs), also referred to as tumor-initiating or tumor-propagating cells. The first experimental evidence supporting the presence of stem cells within human cortical glial tumors was reported in 2002 (Ignatova et al., 2002). Subsequently, numerous studies have corroborated the existence of GSCs in human brain tumors, underscoring their pivotal roles in invasion, angiogenesis, recurrence, and therapeutic resistance (Singh et al., 2003; Singh et al., 2004; Bao et al., 2006a; Bao et al., 2006b; Wakimoto et al., 2009; Cheng et al., 2013). These findings highlight their significance as prime targets for glioblastoma therapy.

## 2 Long non-coding RNAs

Long non-coding RNAs (lncRNAs), typically exceeding 500 nucleotides, are predominantly transcribed by RNA polymerase II and exhibit classical promoter and enhancer elements (Mattick et al., 2023). A significant portion of these lncRNAs undergo processes such as capping, splicing, and polyadenylation, resulting in their characterization as “mRNA-like” (Wang and Chang, 2011; Rinn and Chang, 2012; Mattick et al., 2023). LncRNAs, characterized by cell- or tissue-specific expression, are multifunctional molecules involved in chromatin remodeling, transcriptional regulation, mRNA stabilization, and protein scaffolding, impacting cellular processes like proliferation, differentiation, apoptosis, and metabolism. They also play pivotal roles in developmental processes, immune responses, and the pathogenesis of various diseases, including cancer (Guttman et al., 2010; Guttman et al., 2011; Kung et al., 2013; Long et al., 2017; Yao et al., 2019; Guo et al., 2020; Statello et al., 2021). Recently numerous studies have highlighted their function as molecular sponges, termed competing endogenous RNAs, effectively sequestering microRNAs through competitive interactions with microRNA response elements. This regulatory mechanism modulates gene expression, contributing to the regulation of physiological functions and disease processes (Xiong et al., 2018; Kim et al., 2018; Yang et al., 2022; Zhang et al., 2023a; Wu et al., 2023). In cancer, lncRNAs can act as oncogenes, promoting cancer progression, or conversely, as tumor suppressors, inhibiting tumor growth. Understanding the paradoxical roles that lncRNAs play in cancer biology has paved the way for novel therapeutic strategies, such as silencing oncogenic lncRNAs or restoring the function of tumor-suppressing lncRNAs. Moreover, advances in RNA-based therapeutics, such as antisense oligonucleotides, RNA interference (RNAi), and CRISPR/Cas strategies, offer potential methods for precisely modulating lncRNA activity.

## 3 GSC lncRNAs as oncogenes or tumor suppressors

**FIGURE 1:**
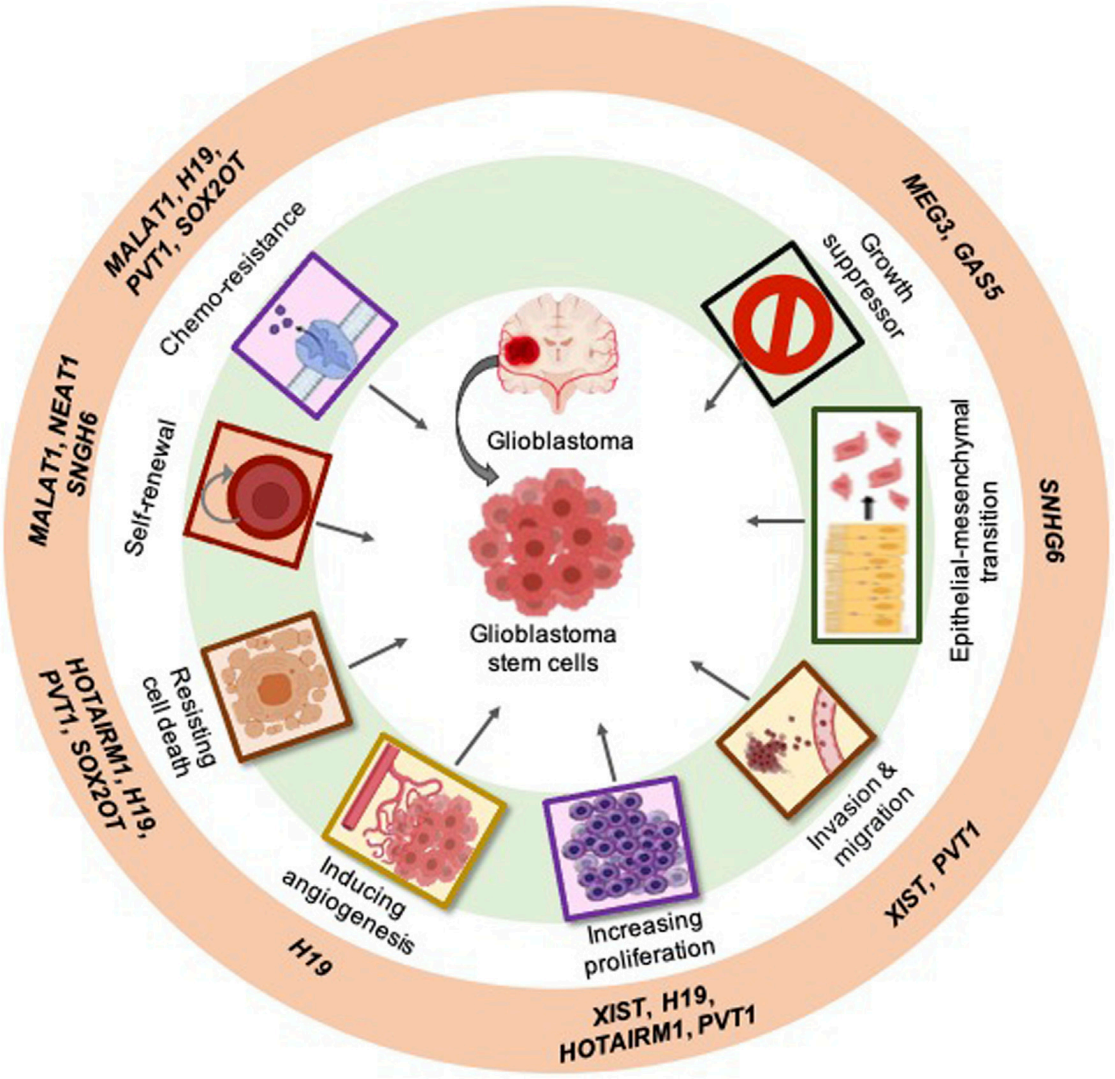
Examples of the most studied long non-coding RNAs (lncRNAs) linked to the hallmark characteristics of cancer within glioblastoma: Examples of the most studied GSC-associated lncRNAs (outer ring). The classical hallmarks of cancer in which lncRNAs play a role (middle ring). lncRNAs can specifically act on GSCs in glioblastoma (center).

Based on clinical data, as well as *in vitro* and *in vivo* evidence, we selected several well-studied examples of lncRNAs involved in the acquired capabilities of GSCs and discussed their regulation in glioblastoma (Figure 1; Table 1).

**TABLE 1:** Exemplary GSC-Associated lncRNAs and Their Regulatory Roles in GSC Biology.

LincRNAs	Proliferation	Self-renewal	Migration/Invasion	Chemoresistance (TMZ)	Mechanism	References
TALNEC2	**↑**	Yes		Yes	TALNEC2 interacts with cell cycle regulatory transcription factor, E2F1 and miR-21 and miR-191	Brodie et al. (2021)
CRNDE	**↑**		**↑**	Yes	CRNDE promotes cell malignant behavior by decreasing miR-384 expression and negative regulation of miR-186	Zheng et al. (2015)
INHEG	**↑**	Yes			Activation of INHEG boosts rRNA 2′-O-methylation, resulting in elevated expression of oncogenic proteins such as EGFR, IGF1R, CDK6, and PDGFRB in GSCs	Liu et al. (2023)
LincRNA-p21		Yes		Yes	The downregulation of lincRNA-p21 suppresses the expression of β-catenin and miR-146b-5p, consequently inducing differentiation in GSCs	Yang et al. (2016), Wang and He (2019)
TUG1		Yes		Yes	TUG1 interacts with EZH2 and inhibits its expression, ultimately reducing TMZ resistance	Cao et al. (2021)
LINC01563	**↑**	Yes			The molecular mechanisms are not known	Hazra et al. (2023)
GIHCG	**↑**	Yes	**↑**		The molecular mechanisms are not known	Hazra et al. (2023)
LUCAT1		Yes			LUCAT1 forms a complex with HIF1α and its co-activator CBP to regulate the expression of HIF1α. Target genes and facilitate GSC adaptation to hypoxia	Huang et al. (2024b)
TP73-AS1				Yes	TP73-AS1 enhances TMZ resistance in GSCs by regulating the cancer stem cell marker ALDH1A1	Mazor et al. (2019)
SNHG1					Hypoxia mediated regulation of SNHG1 in GSCs but detailed mechanism is not known	Gandhi et al. (2023)
FOXD2-AS1	**↑**	Yes			FOXD2-AS1 activates the NOTCH signaling pathway by upregulating TAF-1 and regulates GSC proliferation and stemness	Wang et al. (2022)
LINC01503		Yes			Enhances the GSC properties via reducing FBXW1-mediated proteasomal degradation of GLI2	Wei et al. (2022)
LINC01127		Yes			LINC01127 promotes the self-renewal of GBM cells through the MAP4K4/JNK/NF-κB axis	Li et al. (2023)
LINC02283	**↑**				LINC02283 interacts with PDGFRA to enhance its signaling, along with that of its downstream targets AKT and ERK, promoting oncogenesis in GBM.	Goenka et al. (2023)
GSCAR		Yes		Yes	GSCAR interacts with DHX9-IGF2BP2/SOX2 axis and stabilize SOX2 protein	Jiang et al. (2023)

### 3.1 XIST

*X-inactive-specific transcript* (*XIST*) RNA, discovered in the early 1990s, stands as one of the earliest identified lncRNAs, predating the revelation from the Human Genome Project that a significant portion of our genome comprises noncoding sequences (Brown, 1991; Lander et al., 2001). Despite its original roles in X-chromosome dosage compensation, *XIST* also plays a significant role in regulating cell growth, development, and is implicated in various cancers, including glioblastoma (Yao et al., 2015; Patrat et al., 2020; Yang et al., 2021; Sadagopan et al., 2022). Research has shown that *XIST* is highly expressed in glioma tissues and GSCs. Knockdown of *XIST* significantly reduces cell proliferation, migration, and invasion, while also inducing apoptosis (Yao et al., 2015). Furthermore, *in vivo* studies demonstrate that *XIST* knockdown suppresses tumor growth and improves survival rates in mice (Yao et al., 2015). Another study has demonstrated the involvement of the *XIST*/miR-133a/SOX4 axis, wherein *XIST* interacts with SOX4 and miR-133a, thereby regulating SOX4 expression. This regulatory axis ultimately governs proliferation, migration, metastasis, and the maintenance of glioblastoma stem cells (Luo et al., 2020). In addition, *XIST*, post-transcriptionally regulated by Steroid Receptor Coactivator 1, influences the stemness of glioblastoma cells by modulating the expression of Kruppel-like factor 4 through the *XIST*/miR-152 axis (Gong et al., 2021). Therefore, targeting *XIST* may represent a promising therapeutic approach in glioblastoma.

### 3.2 H19

The imprinted oncofetal lncRNA *H19*, originating from a paternally imprinted gene, is among the earliest identified upregulated lncRNAs observed across various cancer types, including glioblastoma. This lncRNA is expressed during embryonic development, downregulated at birth, and subsequently reappears in tumors (Bartolomei et al., 1991; Raveh et al., 2015; Jiang et al., 2016). One study demonstrated that knocking down *H19* significantly reduces both mRNA and protein levels of GSC markers, such as CD133, NANOG, OCT4, and SOX2 (Li et al., 2016). Additionally, overexpression of *H19* in CD133+ GSCs enhances neurosphere formation and tumor growth (Jiang et al., 2016), suggesting its role in GSC maintenance. Furthermore, *H19* promotes invasion and angiogenesis in glioblastoma, and its overexpression is associated with poor overall survival and progression-free survival (Jiang et al., 2016; Fawzy et al., 2018). Silencing *H19* by TMZ reduces cellular proliferation and increases the apoptosis rate (Li et al., 2016). In addition, *H19* regulates High Mobility Group AT-Hook 2 (HMGA2) expression and microRNAs, both of which enhance the mesenchymal transition of glioblastoma and self-renewal of GSCs. Because *H19* acts as a competing endogenous RNA for microRNAs like let-7, it modulates their availability and indirectly reduces HMGA2 expression (Jiang et al., 2016; Lecerf et al., 2020). This regulatory mechanism orchestrated by *H19* contributes to the maintenance of GSCs and promotes tumor aggressiveness. Hence, *H19* may be a valuable therapeutic target in glioblastoma.

### 3.3 MALAT1

The lncRNA *metastasis-associated lung adenocarcinoma transcript 1* (*MALAT1*) has garnered significant attention as a therapeutic target in various cancers, including glioblastoma. In glioblastoma, *MALAT1* regulates numerous facets of tumor biology, particularly about GSCs, which are pivotal in tumor initiation and progression (Han et al., 2016; Xiong et al., 2018). Moreover, *MALAT1* promotes the self-renewal and maintenance of GSCs by interacting with SOX2 (Williamson, 1989; Xiong et al., 2018; Liao et al., 2019). *MALAT1* modulates key signaling pathways, including the Wnt/β-catenin, Notch, and Hedgehog pathways, which are crucial in GSC biology (Fu et al., 2020). Implicated in mediating therapeutic resistance in GSCs, *MALAT1* confers resistance to chemotherapy, such as TMZ, and radiotherapy—standard treatments for glioblastoma (Chen et al., 2017; Kim et al., 2018). *MALAT1* achieves this by modulating multiple molecular pathways involved in DNA repair, apoptosis, and drug efflux, thus diminishing treatment efficacy and contributing to tumor recurrence and progression (Kim et al., 2018; Baspinar et al., 2020). Strategies aimed at inhibiting *MALAT1* expression or function have shown promising results in preclinical studies, demonstrating the potential for *MALAT1*-targeted therapies to sensitize GSCs to conventional treatments and improve patient outcomes. Further research is necessary to unravel the specific mechanisms by which *MALAT1* regulates GSC behavior and to develop effective *MALAT1*-targeting strategies for treating glioblastoma.

### 3.4 NEAT1

*Nuclear enriched abundant transcript 1* (*NEAT1*) has emerged as a pivotal regulator in GSCs and thus a promising therapeutic target for glioblastoma. Upregulated in GSCs, *NEAT1* contributes to various aspects of tumorigenesis and therapy resistance. *NEAT1* regulates GSC proliferation, self-renewal, stem cell maintenance, and resistance (Bi et al., 2020; Gao et al., 2021; Ghafouri-Fard et al., 2021). Suppressing *NEAT1* impedes the migration and invasion capabilities of glioma cells by modulating the expression of SOX2, targeted by miR-132, underscoring *NEAT1*’s significance in glioma progression and its potential as a therapeutic target (Zhou et al., 2018). Furthermore, *NEAT1* modulates the expression of crucial genes in key signaling pathways, such as Notch, Wnt, and Stat3, which are imperative for GSC maintenance and survival (Zang et al., 2020; Gao et al., 2021; Yuan et al., 2022). By bolstering GSCs and undermining the efficacy of standard treatments, *NEAT1* contributes to tumor progression and unfavorable patient outcomes in glioblastoma (Bi et al., 2020; Gao et al., 2021). Targeting *NEAT1* in GSCs presents an avenue for sensitizing these cells to conventional therapies and enhancing treatment outcomes in glioblastoma.

### 3.5 HOTAIRM1

*HOX antisense intergenic RNA myeloid 1* (*HOTAIRM1*), a transcript previously implicated as a cis-acting factor, is expressed in the myeloid lineage (Zhang et al., 2009) and induced during neuronal differentiation (Lin et al., 2011). Despite its initial identification in myeloid cells, emerging research has revealed its significance as a cancer-related lncRNA, aberrantly expressed in various tumors, including glioblastoma (Zhang et al., 2014; Diaz-Beya et al., 2015; Wan et al., 2016; Li et al., 2018). It is significantly upregulated in GSCs (Li et al., 2018; Xie et al., 2020). Silencing *HOTAIRM1* impairs the proliferation, apoptosis, self-renewal, and tumorigenesis of GSCs, suggesting its crucial role in GSC stemness (Lin et al., 2020; Xie et al., 2020), and thus glioblastoma progression. Additionally, *HOTAIRM1* interacts with EZH2, G9a, and DNA methyltransferases, sequestering them away from the transcription start sites of the HOXA1 gene, thereby activating HOXA1 oncogene expression (Li et al., 2018). Therefore, the oncogenic *HOTAIRM1*/HOXA1 axis in glioblastoma may offer promise as a novel therapeutic target. Another study demonstrated that *HOTAIRM1* promotes cell proliferation and invasion in human glioblastoma by upregulating SP1 via sponging miR-137 (Hao et al., 2020). Furthermore, *HOTAIRM1* has been associated with shorter survival in glioblastoma patients, independent of IDH mutation and O^6^-methylguanine-DNA-methyltransferase promoter methylation (Ahmadov et al., 2021), indicating its role as a driver of biological aggressiveness, radioresistance, and poor outcomes in glioblastoma. Hence, targeting *HOTAIRM1* may represent a promising therapeutic approach in glioblastoma, as it may function as an oncogene.

### 3.6 PVT1

The *plasmacytoma variant translocation 1* (*PVT1*) lncRNA, located on human chromosome 8q24, is co-amplified with MYC and has been identified as an oncogene in several cancers (Tseng and Bagchi, 2015). *PVT1* is upregulated in glioblastoma cells, tissues, and GSCs (Jin et al., 2020; Lv et al., 2022; Hazra et al., 2023). It is associated with TMZ resistance and poor prognosis in glioblastoma patients (Roh et al., 2023; Huang L. et al., 2024). Recent *in vitro* and *in vivo* studies have demonstrated that *PVT1* promotes glioblastoma cell proliferation, invasion, and tumor growth in mice while inhibiting apoptosis (Jin et al., 2020; Lv et al., 2022). In addition, *PVT1* regulates E74-like factor 4 (ELF4) expression by competitively binding to miR-365 and controls the stemness of GSCs via the miR-365/ELF4/SOX2 axis (Gong et al., 2021) Thus, *PVT1* may serve as a potential therapeutic target and prognostic indicator in glioblastoma.

### 3.7 SOX2OT

*SOX2 overlapping transcript* (*SOX2OT*) is highly expressed in embryonic stem cells and upregulated in various cancer types, including glioblastoma (Wang et al., 2020). *SOX2OT* is significantly upregulated in GSCs and glioblastoma tissues, and decreased expression levels of *SOX2OT* impede the malignant biological activities of GSCs by elevating miR-194-5p and miR-122 levels (Su et al., 2017). Another study showed that *SOX2OT* decreases cell apoptosis, enhances cell proliferation, and confers resistance to TMZ by increasing the expression of SOX2, which in turn activates the Wnt5a/β-catenin signaling pathway. These results suggest that *SOX2OT* could potentially serve as a new prognostic biomarker and represent a promising therapeutic target for glioblastoma treatment (Liu et al., 2020).

### 3.8 SNHG6

The lncRNA *small nucleolar RNA host gene 6* (*SNHG6*) has been implicated in various cancer types, yet its role in glioblastoma remains relatively unexplored. Two studies have reported frequent upregulation of *SNHG6* in glioblastoma tissues and cell lines compared to normal brain tissues (Nie et al., 2021; Rahmani et al., 2023), suggesting its involvement in glioblastoma development. Additionally, the downregulation of *SNHG6* in glioblastoma cells induces the expression of the stem cell marker SOX2 and modulates epithelial-mesenchymal transition through interactions with Notch1 and β-catenin (Nie et al., 2021). This transition is critical for tumor metastasis and invasion in glioblastoma. Moreover, high expression levels of *SNHG6* in glioblastoma have been associated with advanced tumor stage, poor prognosis, and shorter overall survival of patients (Nie et al., 2021). Overall, the dysregulation of *SNHG6* in glioblastoma suggests its potential as a diagnostic biomarker and therapeutic target for the disease. However, further research into the precise mechanisms underlying *SNHG6*’s involvement in glioblastoma pathogenesis is warranted to better assess its therapeutic promise.

### 3.9 MEG3

*Maternally expressed gene 3* (*MEG3*), recognized as a tumor suppressor lncRNA, is downregulated across various cancer types, including glioblastoma. In GSCs, this reduced *MEG3* expression corresponds with increased tumorigenicity and stemness (Balci et al., 2016). Restoring *MEG3* function *in vitro* impaired the tumorigenic abilities of GSCs, evidenced by inhibited cell growth, migration, and colony formation, alongside reduced *in vivo* tumor growth, thereby attenuating infiltration. These effects were linked to *MEG3*’s modulation of genes involved in cell adhesion and epithelial-mesenchymal transition (Yang et al., 2020; Leung et al., 2023). Moreover, epigenetic alterations, particularly hypermethylation within the *MEG3*-DMR region, predominantly drive the downregulation of genes within the DLK1-DIO3 locus in glioblastoma (Buccarelli et al., 2020). In addition, decreased expression of *MEG3* is significantly correlated with shorter survival in GBM patients (Jin et al., 2018). Thus, elucidating the functional impact of *MEG3* on GSCs holds significant promise for advancing our understanding of GBM progression and developing targeted therapeutic strategies.

### 3.10 GAS5

*Growth arrest-specific transcript 5* (*GAS5*) is one of the most highly expressed lncRNAs present in human tissues and is implicated in embryogenesis (Hudson et al., 2014). However, its expression is notably reduced across various cancer types, including glioblastoma, and is inversely correlated with clinicopathological characteristics, such as tumor size, staging, or metastasis (Pickard and Williams, 2015). Diminished expression of *GAS5* is observed in high-grade GBM tissues and cells (Zhao et al., 2017), suggesting implications for poorer prognosis. *GAS5* exerts its suppressive effect on GSC malignancy by binding to miR-196a-5p, an onco-miRNA known to promote GSC proliferation, migration, and invasion while reducing apoptosis (Zhao et al., 2017). Moreover, *GAS5* overexpression inhibits cell viability, migration, and invasion in glioblastoma cells, along with impairing stemness and proliferation in GSCs. Furthermore, *GAS5* exhibits anti-oncogenic properties in GBM by modulating the IL-6/STAT3 pathway both *in vitro* and *in vivo*, leading to the inhibition of tumor growth in a xenograft model (Wu et al., 2022). These findings underscore the significant role of *GAS5* in GBM progression, highlighting its potential as a prognostic biomarker and therapeutic target for glioblastoma.

## 4 Experimental and preclinical 3D *in vitro* models to study GSCs

**FIGURE 2:**
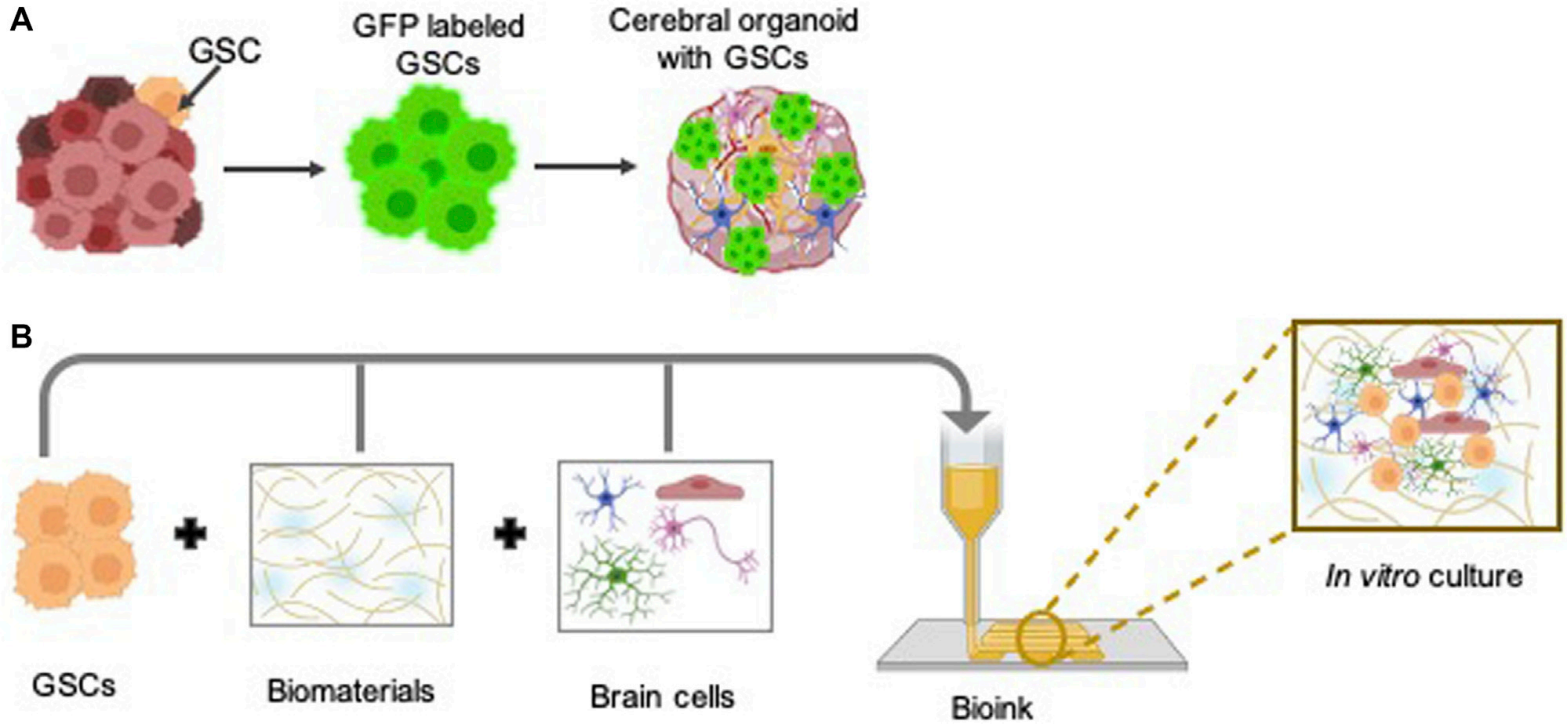
Three-dimensional (3D) preclinical *in vitro* models to study GSC biology: The intricate biology of GSCs within the human brain environment can be effectively explored through the utilization of advanced 3D *in vitro* models, such as glioblastoma cerebral organoids (GLICO) **(A)**, in conjunction with bioprinting techniques **(B)**. Some parts of the figures were created using BioRender.com.

The primary challenge in lncRNA research lies in the limited conservation observed across many lncRNA species. This lack of conservation manifests at the primary sequence, small sequence block, syntenic, and sometimes, structural levels. While certain cancer-associated lncRNAs, such as *MALAT1*, *NEAT1*, and *H19*, show notable conservation, the majority of human cancer-associated lncRNAs lack sequence conservation across mammalian species. This poses a significant barrier to translating findings from human cancer studies to preclinical mouse models. Therefore, alternative models must be explored for preclinical studies aimed at assessing GSC lncRNAs (Figure 2).

Three-dimensional (3D) *in vitro* models are essential for studying GSC biology owing to their capacity to replicate the intricate tumor microenvironment more accurately compared to conventional 2D cultures. Unlike murine models, 3D *in vitro* models provide enhanced throughput, cost-effectiveness, and the ability to mimic human-specific responses, rendering them invaluable for investigating glioblastoma pathophysiology and assessing potential therapeutic interventions. While conventional culture methods offer simplicity, affordability, and speed, they fail to replicate crucial interactions between neural stem cells, perivascular endothelial cells, GSCs, and components of the tumor microenvironment, such as microglia, tumor-activated macrophages, and neurons, which regulate fundamental cancer cell processes like metabolism, proliferation and invasiveness (Bikfalvi et al., 2023). Additionally, GSCs cultured in laminin-adhered 2D monolayers or 3D spheroids in suspension fail to replicate tumor invasion characteristics due to the absence of surrounding matrices. Further, long-term TMZ treatment is impeded by cell overgrowth (Ozturk et al., 2020). Presently, glioblastoma cerebral brain organoids and 3D bio-printed models represent preclinical platforms promising for investigating GSC lncRNAs in glioblastoma.

### 4.1 Glioblastoma cerebral brain organoids (GLICO)

Glioblastoma cerebral brain organoids (GLICO) are innovative approaches for modeling glioblastoma within a physiologically relevant human brain environment. The generation of GLICO entails a multi-step procedure. Firstly, cerebral brain organoids are generated from human embryonic stem cells or fibroblast-derived induced pluripotent stem cells and recapitulate key features of human brain tissue, including its cellular diversity and complex architecture (Lancaster and Knoblich, 2014). Next, GSCs are isolated from freshly obtained patient tumor biopsies (Dundar et al., 2019) or patient-derived xenografts (Hazra et al., 2023) and labeled with a fluorescent marker to facilitate the observation of tumor development within the organoid in real-time. Finally, co-culturing labeled GSCs with cerebral brain organoids (Ogawa et al., 2018; Linkous et al., 2019) or region-specific brain organoids (Fan et al., 2024) presents numerous advantages for comprehensive glioblastoma research. By providing a microenvironment that closely mimics the intricate architecture and cellular interactions of the human brain, cerebral brain organoids offer a physiologically relevant platform to study GSC behavior within a context that mirrors the native tumor niche. Furthermore, these organoids enable researchers to explore GSC responses to various stimuli and therapeutic interventions in a controlled and reproducible manner, thereby facilitating the identification of potential therapeutic targets and the development of personalized treatment strategies for glioblastoma patients. Additionally, by manipulating cerebral brain organoids to replicate the complex tumor microenvironment (TME), characterized by the interplay of immune cells, predominantly microglia, one of the most abundant cellular constituents of the TME, blood vessels, astrocytes, and the extracellular matrix, which exhibits spatial variation across distinct tumor regions, researchers gain valuable insights into the fundamental mechanisms driving glioblastoma initiation and advancement (Bikfalvi et al., 2023). Thus, GLICO models, as advanced preclinical models, hold significant promise for advancing our understanding of glioblastoma pathogenesis, discovering novel therapeutic targets, and evaluating treatment strategies within a biologically relevant context. Furthermore, the GLICO model system provides an unprecedented opportunity to investigate the involvement of GSC lncRNAs by manipulating their expression levels through CRISPR/Cas technology and co-culturing with cerebral organoids, both in the presence and absence of GSC lncRNAs in glioblastoma. However, further research is imperative, given that only three studies have investigated the role of lncRNAs utilizing cerebral brain organoids as model systems in glioblastoma (Liu et al., 2020; Han et al., 2020; Tang et al., 2020). However, one notable disadvantage of cerebral brain organoids is the absence of functional blood vessels, which limits their ability to fully recapitulate the complexity of the tumor microenvironment found *in vivo*. Without a vascular network, organoids lack the physiological perfusion and nutrient exchange seen in actual brain tissue. This deficiency hinders the accurate modeling of aspects such as tumor angiogenesis, drug delivery, and interactions with circulating immune cells. Consequently, while GLICO models offer valuable insights into glioblastoma biology, their utility may be somewhat constrained when studying certain aspects reliant on vascular interactions.

### 4.2 Bioprinting

In pursuit of patient-specific glioblastoma models that faithfully represent the multifaceted complexity of tumors and facilitate accurate therapeutic response assessments, researchers have developed a novel 3D bio-printed glioblastoma model. This innovative model, incorporating patient tumor-derived GSCs, macrophages, astrocytes, neural stem cells, vascular endothelial cells, and decellularized extracellular matrix from brain tissue, accurately recapitulates the structural, biochemical, and biophysical properties of real tumors, encompassing cellular crosstalk, invasion, context-specific functional dependencies, and immunologic interactions within a species-matched neural environment (Yi et al., 2019; Parra-Cantu et al., 2020; Tang et al., 2020). It serves as a dependable platform for exploring drug sensitivity in glioblastoma. Despite significant advancements, traditional 3D bioprinting still faces challenges. However, as this technology continues to evolve, it holds tremendous potential in revolutionizing the combat against brain cancers, offering prospects for enhanced diagnostics, therapies, and ultimately, better patient prognoses. Traditional 3D bioprinting methods are static and may not fully capture the dynamic nature of glioblastoma progression and microenvironmental interactions. Nonetheless, advancements in bioprinting technology, such as 4D, 5D, and even 6D printing (Chadwick et al., 2020; Amiri et al., 2023), present exciting prospects for overcoming these limitations. By introducing temporal and additional spatial dimensions, these advanced models can better mimic the dynamic changes that occur within the tumor microenvironment over time (Yi et al., 2019; Chadwick et al., 2020; Amiri et al., 2023; Yeo et al., 2023). This capability not only improves our understanding of glioblastoma biology but also facilitates the development of more precise and effective therapeutic interventions tailored to individual patient needs.

While 3D bioprinting has been used extensively in various fields of research, including tissue engineering and drug screening, its application to lncRNA study is relatively limited. Given the versatility and precision of 3D bioprinting, researchers could use it to create intricate tissue structures that accurately mimic the *in vivo* microenvironment, facilitating the study of lncRNA functions within their biologically relevant context. Yet despite its promise, further exploration and development are necessary to fully exploit the capabilities of 3D bioprinting for lncRNA research.

## 5 Therapeutic targeting of lncRNAs in glioblastoma

**FIGURE 3:**
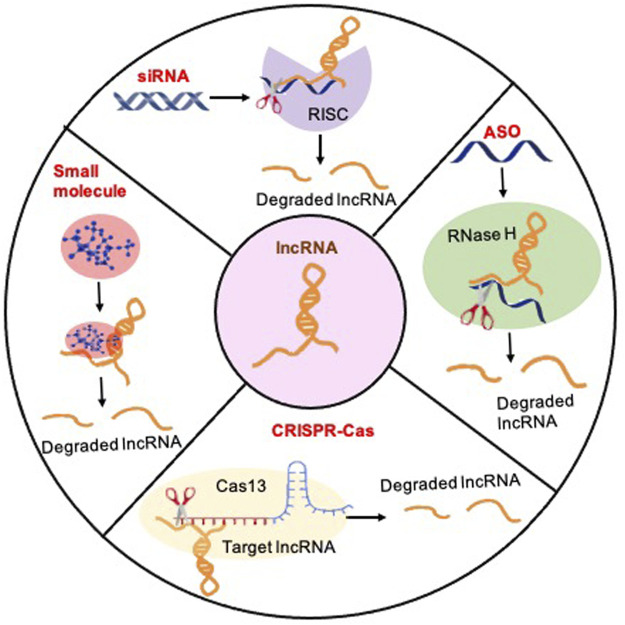
Methods for therapeutically targeting lncRNAs to treat glioblastoma: Four principal therapeutic strategies for lncRNA loss of function: (1) The siRNA-mediated knockdown strategy can be applied to target cytoplasmic lncRNA transcripts by introducing synthetic siRNAs that specifically bind to and degrade the targeted transcripts, thereby effectively suppressing their expression levels. The targeted lncRNA binds to the siRNA-loaded RNA-induced silencing Complex (RISC), which induces the degradation of the lncRNA. (2) The ASO-mediated reduction of nuclear lncRNA transcription levels entails the utilization of antisense oligonucleotides (ASOs) meticulously crafted to bind with nuclear lncRNA transcripts, inducing their degradation or inhibition, consequently diminishing the overall transcriptional activity of the specified lncRNAs within the nucleus. ASOs exploit the cellular enzyme RNase H to catalyze the cleavage of target lncRNAs. (3) CRISPR/Cas strategies represent a potent and accurate approach for targeting lncRNAs. Illustrated here, CRISPR-Cas13 employs a guide RNA to direct the Cas13 protein to designated lncRNA sequences, facilitating the cleavage of the target lncRNA with precision, thereby presenting a promising avenue for precise RNA editing and gene regulation. (4) Small molecules can be directed towards a specific lncRNA to hinder its binding with molecular partners or disrupt proper structure formation.

RNA therapeutics offer a promising avenue for mitigating various ailments, including cancer. Recent strides in genome editing, oligonucleotide chemistry, multi-omics methodologies, and RNA manipulation are streamlining the creation of efficient and economical drug discovery pathways tailored to target lncRNAs. As the exploration in this domain expands, the potential for lncRNA-based therapeutics to revolutionize cancer management has become increasingly apparent. Promising therapeutic strategies to target lncRNAs include small interfering RNAs (siRNAs), antisense oligonucleotides (ASOs), CRISPR gene editing, and small molecules that recognize RNA structures (Figure 3).

### 5.1 Small interfering RNAs (siRNAs)

Ranging from 19 to 30 nucleotides in length, siRNAs are short double-stranded RNAs that initiate gene silencing by engaging the RNA-induced silencing complex to degrade the target gene through posttranscriptional mechanisms (Hannon and Rossi, 2004). The therapeutic potential of siRNAs in human disease spans a wide spectrum, including applications in genetic disorders, viral infections, and neurodegenerative diseases (Gavrilov and Saltzman, 2012). Several therapeutic drugs utilizing siRNAs have been developed and are either available on the market or in clinical trials. Notably, patisiran (ONPATTRO™) harnesses siRNA technology to treat hereditary transthyretin-mediated amyloidosis (Coelho et al., 2013; Hoy, 2018; Urits et al., 2020). By targeting and reducing the production of abnormal transthyretin protein, patisiran effectively mitigates disease progression, showcasing the potential of siRNA-based therapies. While there are currently no FDA-approved siRNA-based therapeutic drugs for cancer, many are in various stages of preclinical and clinical development. For instance, systemic delivery of *MALAT1* siRNA via a nano complex that targets GSCs significantly reduced the growth, infiltration, and stemness of GSCs improved the sensitivity of GSCs to TMZ, and increased the survival of animals (Kim et al., 2018). This suggests that *MALAT1* could serve as a preclinical model for glioblastoma. Although lncRNA-based siRNA therapeutics hold promise for GBM treatment, there are concerns regarding their clinical applications. One challenge is efficiently delivering siRNA molecules across the blood-brain barrier and into tumor cells within the brain. Additionally, off-target effects and the potential immune stimulation associated with siRNA therapy may pose risks to healthy tissues and compromise the overall safety of the treatment. Lastly, the development of resistance to siRNA-mediated gene silencing could limit the long-term efficacy of this therapeutic approach.

### 5.2 Antisense oligonucleotides (ASOs)

ASOs are synthetic single-stranded DNA or RNA molecules, typically 15–21 nucleotides in length, specifically engineered to bind to complementary RNA sequences through standard Watson-Crick base pairing mechanisms (Crooke et al., 2021). This binding capability allows ASOs to exert their effects by modulating gene expression at the posttranscriptional level. ASOs have emerged as powerful tools for manipulating gene expression, including the knockdown of lncRNAs (Lang et al., 2021; Adewunmi et al., 2023). Various types of ASO modifications, such as phosphorothioate (PS), 2′-methoxyethyl (2′-MOE), 2′-constrained ethyl (2′-cEt), and locked nucleic acid, have been developed to enhance their efficacy and specificity in RNA knockdown (Crooke et al., 2021). A recent study demonstrated the promising preclinical therapeutic target of a high-grade pediatric glioma known as diffuse intrinsic pontine glioma (DIPG) using 2′-MOE ASO (Zhang et al., 2023b). The hallmark genetic alteration of DIPG is a mutation in the genes encoding histone H3.3, which results in a lysine-to-methionine substitution at position 27 (K27M); this alteration is found in approximately 80% of DIPG cases (Wan et al., 2018). The 2′-MOE-PS ASOs efficiently degrade H3-3AK27M mRNA, resulting in reduced levels of H3.3K27M protein in both *in vitro* and *in vivo* systems (Zhang et al., 2023b). These findings suggest the potential of ASO therapy as a viable treatment option for DIPG (Zhang et al., 2023b). In glioblastoma, intravenous treatment with ASOs targeting *TUG1*, in conjunction with a sophisticated drug delivery system, induces GSC differentiation and efficiently inhibits GSC growth *in vivo*, indicating that TUG1 may be a potent therapeutic approach to eradicate the GSC population (Katsushima et al., 2016). Another preclinical study demonstrated that targeting *MALAT1* with modified ASOs promotes the differentiation of tumor cells and decreases tumor growth in a breast cancer mouse model (Arun et al., 2016)—a strategy that may be suitable for other solid cancers, including glioblastoma. These examples highlight the potential of ASOs and lncRNA-targeted therapies for precise and targeted intervention in glioblastoma, offering new avenues for therapeutic development and personalized treatment strategies.

### 5.3 CRISPR-Cas system

The advent of CRISPR-Cas systems has effectively mitigated the limitations inherent in RNAi for identifying cancer-promoting lncRNAs (Meira et al., 2021). CRISPR technology facilitates the precise perturbation of lncRNA expression in both the nucleus and cytoplasm, enabling reversible or permanent modifications (Li et al., 2020). Furthermore, CRISPR offers scalability for high-throughput assays, both *in vitro* and *in vivo*, owing to its compatibility with pooled screening formats (Bock et al., 2022). This scalability obviates the need for costly arrayed libraries or screening robots, as CRISPR-based assays yield direct functional readouts and adaptable targeting libraries, regularly updated to incorporate evolving gene annotations (Bock et al., 2022). Nevertheless, generating loss-of-function alleles for lncRNAs with a conventional CRISPR-Cas9 system is slightly more intricate than for protein-coding genes due to lncRNA’s inherent lack of long open reading frames. To overcome this challenge, a dual guide RNA approach is employed, facilitating the simultaneous creation of two DNA double-strand breaks, potentially resulting in the deletion of the entire lncRNA gene or its promoter region, a technique known as CRISPR deletion (Hazra et al., 2022). CRISPR technology can manipulate lncRNA genes without requiring DNA mutations. Using CRISPR interference (CRISPRi), a recent study identified lncGRS-1 as a glioma-specific therapeutic target. Knockdown of lncGRS-1 effectively impedes the proliferation of primary adult and pediatric glioma cells while preserving the integrity of normal brain cells (Liu et al., 2020). This finding underscores the utility of a swift and precise methodology for identifying promising therapeutic targets within the non-coding genome, thereby advancing the potential of radiation therapy in glioma treatment (Liu et al., 2020). Furthermore, CRISPR can be harnessed to directly target RNA using systems like single-stranded RNA-binding CRISPR-Cas13. This approach exhibits knockdown efficiency comparable to RNAi but with significantly reduced off-target effects (Abudayyeh et al., 2017). Recently, A Cas13d/CasRx-based screening platform was developed for genome-scale interrogation of lncRNAs (Montero et al., 2024), which overcomes the limitations observed in other lncRNA perturbation approaches, such as low targeting efficiency, off-target effects, and inadvertent perturbation of other DNA elements. The system has been further refined to enhance its applicability across various cancer types, enabling the systematic exploration of lncRNA biology, identification of context-specific traits, and discovery of lncRNA vulnerabilities suitable for therapeutic targeting (Montero et al., 2024). This study highlights that CasRx-based lncRNA screening effectively addresses major limitations associated with alternative perturbation methods. For example, RNAi is hindered by low nuclear activity and high off-target rates, whereas CasRx, fused with a nuclear localization signal, demonstrates robust nuclear activity and precise targeting of lncRNAs. Notably, CasRx exhibits high specificity in targeting lncRNAs, with the optimal guide RNA length ranging from 20 to 23 nucleotides, compared to the 2- to 7-nucleotide seed region typical of siRNAs (Konermann et al., 2018; Li et al., 2021). Taking into account all of these advantages, along with the potential for RNA-degrading drugs, CRISPR-CasRx may emerge as the preferred option for targeting lncRNAs.

### 5.4 Small molecules

Targeting lncRNAs with small molecules or natural compounds represents a promising therapeutic approach for glioblastoma. For example, small molecule inhibitors can target the lncRNA HOTAIR, recognized for its role in promoting metastasis through chromatin remodeling and gene expression regulation (Li et al., 2019). In a notable preclinical study, targeting the MALAT1 ENE (Exonuclease Protection Element) triplex with small molecules holds great promise for developing novel anticancer therapies and molecular tools to investigate MALAT1-driven cancers. The MALAT1 ENE triplex, a specific structural motif within the MALAT1 RNA molecule, is known to play a crucial role in cancer progression and metastasis. Disruption of this motif has the potential to inhibit cancer cell growth and metastasis, offering new avenues for cancer treatment. Additionally, these small molecules serve as valuable molecular probes in research settings, allowing for precise study of the mechanisms underlying MALAT1-driven cancers (Abulwerdi et al., 2019). These endeavors underscore the growing significance of small molecule-based approaches in modulating lncRNA function, offering innovative avenues for effective cancer therapies targeting these non-coding transcripts. Understanding higher-order RNA structures is imperative for designing small molecules targeting lncRNAs (Childs-Disney et al., 2022). While the feasibility of targeting RNAs with small molecules has been explored with some success, a deeper comprehension of RNA-ligand interactions, additional RNA targets, and active compounds targeting RNAs is necessary for further advancements.

## 6 Conclusion

Embarking on advancements in radiotherapy, surgery, and contemporary medical modalities, the challenge of glioblastoma persists as a significant concern due to its high mortality rates. Exploring the potential synergy between GSC lncRNAs and emerging therapies offers hope for enhancing treatment outcomes, yet demands a profound mechanistic comprehension of their roles in glioblastoma. Studies have unveiled associations between specific lncRNA expression and glioblastoma malignancy grade, histological differentiation, and diagnosis (Zhang et al., 2012). While harnessing lncRNAs for novel diagnostics and therapeutics in glioblastoma holds promise, many aspects of lncRNA biology remain elusive. Therefore, employing lncRNAs for diagnostic and therapeutic purposes without a comprehensive grasp of their multifaceted functions may be premature.

The detailed characterization of each lncRNA and the development of novel therapeutics for glioblastoma present significant challenges. Nevertheless, recognizing lncRNAs as crucial contributors to tumorigenesis provides an opportunity to explore their expression patterns and mechanisms of action for targeted cancer therapies. Moreover, the rapid progress in sequence-based nucleic acid therapeutics offers avenues for exploiting lncRNAs as therapeutic targets in cancer treatment. Therefore, identifying and evaluating lncRNA targets within the glioblastoma context represent vital steps toward translating research insights into clinical applications.
